# Supplementary material for: Rate of Malignant Transformation Differs Based on Diagnostic Criteria for Oral Lichenoid Conditions: A Systematic Review and Meta-Analysis of 24,277 Patients
Source: Cancers (Basel). 2023 Apr 28;15(9):2537. doi: 10.3390/cancers15092537 (PMC10177058; doi:10.3390/cancers15092537)
Supplement: Supplementary file 1 [file cancers-15-02537-s001.zip › cancers-2340713-supplementary.pdf]

Supplementary Tables and Figures

Rate of Malignant Transformation Differs Based on Diagnostic Criteria for Oral Lichenoid Conditions: A Systematic Review and Meta-Analysis of 24,277 Patients

Jing-Wen Li [1], Kar Yan Li [2], Bik Wan Amy Chan [3], Colman Patrick McGrath [4] and Li-Wu Zheng [1,*]

[1] Division of Oral & Maxillofacial Surgery, Faculty of Dentistry, The University of Hong Kong, Hong Kong SAR, China; jingwen7883@gmail.com
[2] Clinical Research Centre, Faculty of Dentistry, The University of Hong Kong, Hong Kong SAR, China; skyli@hku.hk
[3] Department of Anatomical and Cellular Pathology, Prince of Wales Hospital, The Chinese University of Hong Kong, Hong Kong SAR, China; abwchan@cuhk.edu.hk
[4] Division of Applied Oral Sciences & Community Dental Care, Faculty of Dentistry, The University of Hong Kong, Hong Kong SAR, China; mcgrathc@hkucc.hku.hk
* Correspondence: lwzheng@hku.hk; Tel.: +852-28592558

Table S1. Baseline data and characteristics of the included studies. (excel document).

R: retrospective; **P**:prospective; OLP: oral lichen planus; OLL: oral lichenoid lesions; LMD: lichenoid mucositis with dysplasia; OSCC: Oral squamous cell carcinoma; MT: malignant transformation; M:male; F: female; P: positive; N: negative; "/": not mentioned

Table S2. Risk of bias assessment using the modified Newcastle-Ottawa scale.

**Study; Year**	**Selection**			**Comparability**	**Outcomes**				**Modified Newcastle-Ottawa score (risk of bias)**
	Representativeness of an exposed cohort	Diagnosis based on both clinical and histopathological confirmation	Well differentiated of OLP and epithelial dysplasia	Consider documenting any carcinogenic factors while analysing/reporting the outcomes	Assessment of outcome	Details information about malignant transformation	Sufficient follow-up duration (2 years or more)	Adequacy of Follow Up of Cohorts	
Van der Meij et al.;2003	✓	✓	✓		✓	✓		✓	**6**
Machado et al.; 2004	✓			✓	✓	✓		✓	**5**
Rödstrom et al.; 2004	✓				✓	✓		✓	**4**
Mattila et al.; 2004	✓	✓			✓	✓		✓	**5**
Gandolfo et al.;2004	✓			✓	✓	✓		✓	**5**
Xue et al.;2005	✓	✓	✓	✓	✓	✓		✓	**7**
Laeijendecker et al.;2005	✓	✓		✓	✓	✓	✓	✓	**7**
Roosaar et al.;2006	✓			✓	✓	✓		✓	**5**
Bornstein et al.;2006	✓	✓	✓	✓	✓	✓		✓	**7**
Ingafou et al.;2006	✓	✓			✓	✓		✓	**5**
Hsue et al.;2007	✓	✓			✓			✓	**4**
Van der Meij et al.;2007	✓	✓	✓	✓	✓	✓		✓	**7**
Kesic et al.;2009	✓	✓		✓	✓			✓	**5**
Fang et al.;2009	✓	✓	✓	✓	✓	✓		✓	**7**
Pakfetrat et al.;2009	✓	✓		✓	✓	✓		✓	**6**
Ögmundsdóttir et al.; 2009	✓	✓			✓		✓	✓	**5**
Carbone et al.;2009	✓	✓	✓	✓	✓	✓		✓	**7**

Oliveira et al.;2010	✓	✓		✓	✓			✓	**5**
Zyada et al.;2010	✓	✓		✓	✓	✓	✓	✓	**7**
Thongprasom et al.;2010	✓			✓	✓	✓	✓	✓	**6**
Bajaj et al.;2010	✓		✓	✓	✓			✓	**5**
Bermejo-Fenoll et al.;2010	✓	✓		✓	✓			✓	**5**
Torrente-Castells et al.;2010	✓	✓		✓	✓	✓		✓	**6**
Bombeccari et al.;2011	✓	✓	✓	✓	✓	✓		✓	**7**
Jaafari-Ashkavandi et al.;2011	✓				✓	✓		✓	**4**
Warnakulasuriya et al.;2011	✓	✓			✓			✓	**4**
Brzak *et al.,* 2012	✓				✓			✓	**3**
Kaplan *et al.,* 2012	✓	✓		✓	✓	✓		✓	**6**
Shen *et al.,* 2012	✓	✓		✓	✓	✓	✓	✓	**7**
Bardellini et al., 2013	✓	✓	✓	✓	✓	✓		✓	**7**
Gümrü *et al.*, 2013	✓	✓	✓	✓	✓	✓		✓	**7**
Tovaru *et al.* 2013	✓	✓			✓	✓		✓	**5**
Budimir *et al.,* 2014	✓			✓	✓		✓	✓	**5**
Radochová et al. 2014	✓	✓	✓	✓	✓	✓		✓	**7**
Wang et al. 2014	✓	✓	✓	✓	✓			✓	**6**
Casparis *et al.*, 2015	✓	✓	✓		✓	✓		✓	**6**
Mostafa et al. 2015	✓		✓	✓	✓			✓	**5**
Lauritano *et al.*, 2016	✓	✓	✓		✓			✓	**5**
Irani *et al.,* 2016	✓	✓			✓			✓	**4**
Varghese et al. 2016	✓	✓	✓	✓	✓			✓	**6**

Yahalom et al. 2016	✓	✓		✓	✓			✓	**5**
Bandyopadhyay *et al.,* 2017	✓	✓			✓	✓		✓	**5**
Gonzalez-Moles *et al.*, 2017	✓	✓			✓			✓	**4**
Rimkevičius et al., 2017	✓	✓			✓			✓	**4**
Park et al. 2018	✓		✓		✓			✓	**4**
Rock et al. 2018	✓	✓	✓	✓	✓			✓	**6**
Laniosz et al., 2018	✓			✓	✓			✓	**4**
Shearston *et al.,* 2019	✓	✓	✓		✓	✓		✓	**6**
Guan et al. 2020	✓	✓	✓	✓	✓	✓		✓	**7**
Arduino et al. 2021	✓	✓	✓	✓	✓			✓	**6**
Radochová et al. 2021	✓	✓		✓	✓	✓		✓	**6**
Tsushima et al. 2021	✓	✓	✓	✓	✓	✓		✓	**7**
Zotti et al. 2021	✓	✓	✓	✓	✓	✓	✓	✓	**8**
Cai et al. 2022	✓	✓	✓	✓	✓	✓		✓	**7**

Table S3. Evaluation of quality of evidence using Grading of Recommendations Assessment, Development and Evaluation (GRADE) system.

№ of studies	Certainty assessment						Effect			Certainty
	Study design	Risk of bias	Inconsistency	Indirectness	Imprecision	Other considerations	№ of MT events	№ of patients	Rate (95% CI)	
**OLP malignant transformation**										
51	observational studies	serious	Serious $I^2 = 67.30\%$ $p = .000$	not serious	not serious	strong association	283	22578	event rate 1.1% (0.82 to 1.32)	⊕⊕⊕◯ Moderate
**OLL malignant transformation**										
6	observational studies	not serious	Serious $I^2 = 52.10\%$ $p = .064$	not serious	not serious	none	14	717	event rate 1.9% (0.15 to 3.75)	⊕⊕⊕◯ Moderate
**LMD malignant transformation**										
4	observational studies	not serious	Not serious $I^2 = 0.0\%$ $p = .488$	not serious	not serious	none	11	153	event rate 6.3% (2.3 to 10.32)	⊕⊕⊕⊕ High

Certainty assessment							№ of patients		Effect		Certainty
№ of studies	Study design	Risk of bias	Inconsistency	Indirectness	Imprecision	Other considerations	[intervention]	[comparison]	Relative (95% CI)	Absolute (95% CI)	
**[intervention]: Smokers ; [comparison]: non-Smokers**											
14	observational studies	not serious	not serious<br>I^2 = 20.60%<br>p = .195	not serious	Serious<br>(Precise dose and frequency were not available for individual studies)	strong association	27/1317 (2.1%)	112/6277 (1.8%)	**OR 1.79** (1.02 to 3.03)	**14 more per 1,000** (from 0 fewer to 34 more)	⊕⊕◯◯ Low
**[intervention]: alcohol consumption ; [comparison]: non-alcohol consumption**											

Certainty assessment							№ of patients		Effect		Certainty
№ of studies	Study design	Risk of bias	Inconsistency	Indirectness	Imprecision	Other considerations	[intervention]	[comparison]	Relative (95% CI)	Absolute (95% CI)	
7	observational studies	not serious	not serious<br>$I^2 = 23.00\%$<br>$p = .254$	not serious	Serious<br>(Precise dose and frequency were not available for individual studies)	strong association	8/397 (2.0%)	21/2378 (0.9%)	**OR 3.27** (1.11 to 9.64)	**19 more per 1,000** (from 1 more to 70 more)	⊕⊕◯◯ Low
**[intervention]: HCV-positive ; [comparison]: HCV-negative**											
10	observational studies	not serious	not serious<br>$I^2 = 0.00\%$<br>$p = .536$	not serious	not serious	strong association	25/644 (3.9%)	110/6206 (1.8%)	**OR 2.55** (1.58 to 4.13)	**26 more per 1,000** (from 10 more to 52 more)	⊕⊕⊕◯ Moderate
**[intervention]: diabetes mellitus ; [comparison]: non-diabetes mellitus**											

Certainty assessment							№ of patients		Effect		Certainty
№ of studies	Study design	Risk of bias	Inconsistency	Indirectness	Imprecision	Other considerations	[intervention]	[comparison]	Relative (95% CI)	Absolute (95% CI)	
8	observational studies	not serious	not serious<br>$I^2 = 0.00\%$<br>p = .955	not serious	not serious	strong association	4/340 (1.2%)	36/3311 (1.1%)	**OR 1.89** (0.80 to 4.44)	**9 more per 1,000** (from 2 fewer to 36 more)	⊕⊕⊕◯ Moderate
**[intervention]: hypertension ; [comparison]: non-hypertension**											
5	observational studies	not serious	Serious<br>$I^2 = 76.7\%$<br>p = .002	not serious	not serious	strong association	7/423 (1.7%)	31/2169 (1.4%)	**OR 1.48** (0.22 to 10.25)	**7 more per 1,000** (from 11 fewer to 115 more)	⊕⊕◯◯ Low
**[intervention]: male ; [comparison]: female**											

Certainty assessment							№ of patients		Effect		Certainty
№ of studies	Study design	Risk of bias	Inconsistency	Indirectness	Imprecision	Other considerations	[intervention]	[comparison]	Relative (95% CI)	Absolute (95% CI)	
31	observational studies	not serious	not serious $I^2 = 0.00\%$ $p = .905$	not serious	not serious	very strong association	89/6701 (1.3%)	144/12687 (1.1%)	**OR 1.18** (0.90 to 1.55)	**2 more per 1,000** (from 1 fewer to 6 more)	⊕⊕⊕⊕ High
**[intervention]: clinical pattern-red ; [comparison]: clinical pattern-white**											
21	observational studies	not serious	not serious $I^2 = 20.60\%$ $p = .195$	not serious	not serious	very strong association	111/4382 (2.5%)	61/6755 (0.9%)	**OR 3.52** (2.20 to 5.64)	**22 more per 1,000** (from 11 more to 40 more)	⊕⊕⊕⊕ High

An initial baseline overall quality of "very low quality"(for observational study) of evidence was assigned to the outcome under analysis. Then, this rating is "upgraded" based on the following domains: risk of bias, inconsistency, indirectness, and imprecision (overall quality of evidence rating was not "upgraded" according to any criteria, for example, magnitude of effect size). The quality of evidence is classified in one of four levels: very low, low, moderate, or high. A "serious" score downgrades one level of evidence, "very serious" two levels. Although "large effect size" upgrades one level of evidence, no variables were considered for upgrading. Risk of bias: Quality was assessed according to the respective risk of bias analyses performed by each systematic review (i.e., QUIPS tool). Inconsistency: Heterogeneity was assessed via Q test and I^2 statistic. I^2 >50% and/or Q test p-values ≤.10 were considered as significant heterogeneity and a "serious" rating was assigned. Indirectness: According to our judgment and knowledge, all outcomes were considered as sources of direct evidence (i.e., a research that directly

compares the exposures which we are interested in, target subpopulations and outcomes of interest). Imprecision: If wide confidence intervals and/or small sample sizes ("rule of thumb" ≤10 studies) were present, a "serious" rating was assigned. Publication bias: If publication bias was strongly suspected, it was recorded for descriptive purposes. Nevertheless, due to the lack of consensus on the influence of publication bias in meta-analyses of proportions, this domain was not rated nor considered for downgrading. GRADE certainty ratings. Very low: The true effect is probably markedly different from the estimated effect; Low: The true effect might be markedly different from the estimated effect; Moderate: The authors believe that the true effect is probably close to the estimated effect; High: The authors have a lot of confidence that the true effect is similar to the estimated effect.

Abbreviations: CI, confidence intervals; MT, malignant transformation; OLP, oral lichen planus; OLL, oral lichenoid lesions; LMD, lichenoid mucositis dysplasia

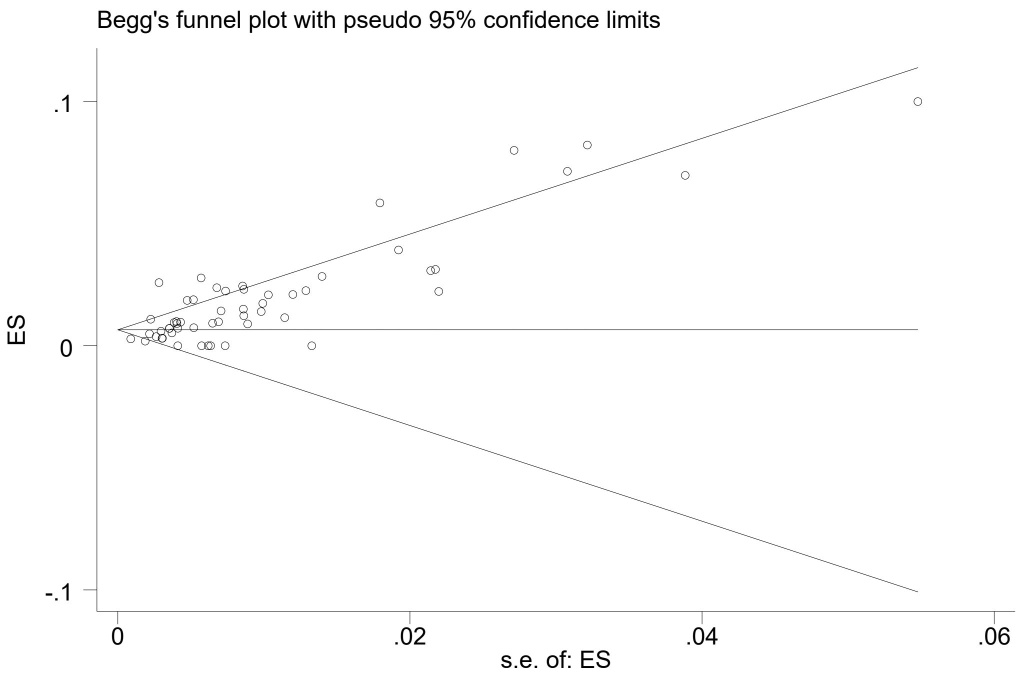

Figure S1. Funnel plot of publication bias for all included studies.

Egger's test

Std_Eff	Coef.	Std. Err.	t	P>|t|	[95% Conf. Interval]	
slope	.0016946	.0011366	1.49	0.142	-.0005863	.0039754
bias	1.705538	.2962487	5.76	0.000	1.111071	2.300004
